# Exploring the Brine Microbiota of a Traditional Norwegian Fermented Fish Product (*Rakfisk*) from Six Different Producers during Two Consecutive Seasonal Productions

**DOI:** 10.3390/foods8020072

**Published:** 2019-02-14

**Authors:** Guro Alette Bjerke, Knut Rudi, Ekaterina Avershina, Birgitte Moen, Hans Blom, Lars Axelsson

**Affiliations:** 1 Nofima, Norwegian Institute of Food, Fisheries and Aquaculture Research, P.O. Box 210, NO-1431 Ås, Norway; gurbje@gmail.com (G.A.B.); birgitte.moen@nofima.no (B.M.); hans.blom.personlig@gmail.com (H.B.); 2 Department of Chemistry, Biotechnology and Food Sciences, Norwegian University of Life Sciences, P.O. Box 5003, N-1432 Ås, Norway; knut.rudi@nmbu.no (K.R.); ekaterina.avershina@nmbu.no (E.A.)

**Keywords:** fermented fish, lactic acid bacteria, *Gammaproteobacteria*, microbiota, sequencing, ripening conditions

## Abstract

The purpose of this study was to explore the microbiota of Norwegian fermented fish (*rakfisk*), a traditional product popular in the Norwegian market. Brine samples, collected from six producers during two subsequent years, were used. The producers applied different salt concentrations (between 3.8% and 7.2% NaCl), ripening temperatures (between 3.5 and 7.5 °C), fish species (trout or char), and fish upbringing (wild trout, on-shore farmed trout or char, and off-shore farmed char). The microbiota in the brine during the ripening process was mainly characterized by DNA-based, culture-independent methods. In total, 1710 samples were processed and of these 1342 were used for the final analysis. The microbiota was dominated by *Gammaproteobacteria* and *Bacilli* with the largest variance between samples associated with the genera *Psychrobacter* and *Lactobacillus*. The variance in the material was mainly determined by the origin of the samples, i.e., the different producers. The microbiota from the individual producers was to a large extent reproducible from one year to the next and appeared to be determined by the relatively small differences in the salinity and the ripening temperature. This is the first study exploring the microbiota in *rakfisk* brine and it provides insights into environmental factors affecting the *rakfisk* ecosystems.

## 1. Introduction

Fermentation is an ancient processing technique that not only makes it possible to preserve the food, but also alters attributes like flavor, odor and texture in a way that consumers appreciate [1,2]. These features arise from activity of microorganisms and/or endogenous enzymes when proteins, polysaccharides and lipids are hydrolyzed and sugars, amino acids and fatty acids are converted to fermentation end products and aroma compounds [3]. In many fermented foods, lactic acid bacteria (LAB) often become dominant and largely contribute to the fermentation [2,4,5]. LAB has also been found to be dominant in fish and other seafood fermentations [6,7,8,9,10,11]. 

Norwegian fermented fish (*rakfisk*) is a traditional product with an increasing popularity in the Norwegian market (about 400 tons traded per year). *Rakfisk* is manufactured in a traditional, artisanal, and localized manner and is known for its characteristic taste, odor, and somewhat spreadable texture. Use of the name *rakfisk* can be traced back to the middle ages, but the practice of making the product is probably even older as it shares similarities with other ancient food fermentation techniques [12]. The manufacture of *rakfisk* is based on mild salting of salmonid freshwater fish, mainly lake trout and some arctic char. In the most commonly used procedure, the gutted fish is dry salted and layered (belly up), preferably under pressure, in tight containers. A few producers may add a small amount of sugar and/or a LAB-based starter culture together with the salt. Spontaneous brining occurs quickly after dry salting. Alternatively, premade brine is used by some producers for salting. In both cases, the fish is submerged in the salt brine during the storage/maturation period. Salt concentration in the brine (and in the fish after equilibration) is approximately 4–7% (*w/w*), depending on the producer. The containers are stored at low temperatures (3–8 °C) for 3–12 months, during which a fermentation/ripening takes place [12,13]. The product is generally consumed without any heat treatment and has therefore been subject to food safety concerns [12]. With the exception of very old work [14], and despite the popularity in the Norwegian market, *rakfisk* has not been the subject of proper scientific investigations. So far, only preliminary studies and anecdotal observations have been made pertaining to the microbiological development and the product being the result of a lactic acid fermentation [12]. Globally, few fermented fish products share similarities in the manufacture process with *rakfisk*, at least described in the scientific literature. However, *sikhae* (Korea) [15], *Suan Yu* (China) [10], and *Chouguiyu* (China) [6,16] are all mildly salted fish, fermented in brine at relatively low temperatures. All these fermentations seem to be dominated by salt- and cold-tolerant lactobacilli, in some cases determined to be *Lactobacillus sakei* [6,15].

Hypervariable regions in the 16S ribosomal RNA (rRNA) gene are the most common target for analyzing diversity in a variety of complex microbial communities, including food [17], with several examples from seafood fermentations [7,15,18].

The aim of this study was to characterize the microbiota in *rakfisk* fermentation. For logistic reasons, *rakfisk* brine was used for sampling. Brine was collected during the course of production and maturation from six producers using different salt concentrations, ripening temperatures, fish upbringing, and species. This is the first study of *rakfisk* microbiota and, to our knowledge, the first study of the microbiota of any fermented fish product where samples were collected from the same producers for two consecutive seasonal productions. We used a 16S rRNA gene direct dideoxy sequencing approach [14] to screen for the dominating microbiota and a selection of samples were sequenced (16S rDNA V4) using the Illumina (MiSeq) methodology to verify the direct dideoxy sequencing results and to get a more in-depth view of the microbiota. The total number of bacteria was estimated in a culture-independent manner using quantitative real-time polymerase chain reaction (PCR). As complements to the main study, bacteria were also quantified using plating on various agar media and a number of LAB isolates were subjected to 16S rRNA gene sequencing to determine the species.

## 2. Materials and Methods

### 2.1. Production Conditions

In this project, brine collected during the *rakfisk* ripening process was analyzed. Six individual producers, using different fish species, origin of the fish, ripening temperatures, salt concentrations and additions (Table 1), participated in the project by collecting brine samples from own productions during two subsequent production years.

An additional experiment in which Producer 1 changed the normal ripening conditions of one separate batch was also included (Table 1).

### 2.2. Experimental Design and Sampling

The freshly slaughtered fish was gutted and cleaned. Five of the producers prepared the fish in a traditional dry salting manner where the fish was tightly packed in closed plastic containers. Brine forms naturally during the first day, but in case the brine did not completely cover the fish after the first 24 h, the containers were topped up with brine holding the appropriate salinity. The last producer placed the fish directly in containers with premade brine (Table 1). Producer 1 used 40-liter containers (approximately 36 kg fish per container); Producer 6 used 30-liter containers (approximately 27 kg fish per container), while the other 4 producers used 10-liter containers (approximately 9 kg fish per container) for the experiment. The fish was submerged in brine for the whole 91 days ripening period and the containers were stored in cold rooms. Brine was collected aseptically by the producers themselves (provided with utensils and equipment from the research facility) in three technical parallels from the top-, the middle- and the bottom layers of the containers the first year and from the top- and middle layer the following year. In order to ease the sampling and to be able to collect brine from the middle and the bottom layer without moving the fish, the containers/buckets were equipped with a pipe placed vertically in the center through which the samples were withdrawn. The pipe was about 10 cm in diameter, perforated throughout with holes of one square cm and slightly shorter that the height of the containers so that the lids could be properly closed. The samples were then frozen at −19 °C until analysis. The Producers 1, 2 and 3 collected brine samples at days 0, 3, 7, 14, 28, 42, 63, and 91 (i.e., full sampling series; 72 samples per producer per year) during two production years. Producers 4, 5 and 6 collected brine samples only at the end of the ripening period the first year, but performed the complete sampling series the second year. The day 0 samples were collected the day after the fish had been placed in the containers (i.e., after brining had occurred), with the exception of Producer 6. This producer had a different manufacturing procedure since the gillnet fishing, the preparation of the fish and the first ripening period took place in the mountain area, where the chill storing conditions were suboptimal, i.e., the temperature was around 9–10 °C. To compensate for a higher and less controlled ripening temperature during the mountain storage, a salt concentration of about 12% NaCl were used. After approximately 10 days, the fish containers were moved to the producer’s premises in the village and the brine was diluted to 7.2% NaCl. Due to lack of a freezer compartment during the mountain storage, the sample collection started the day the fish containers were relocated to the village (i.e., the day 0 samples from Producer 6 contained brine from *rakfisk* ripened for 10 days in high salt concentration). In addition, the final samples from Producer 6 were collected on day 63, since the *rakfisk* at that point was ready to be consumed and was sold. Each producer had three containers especially assigned for sampling each year.

### 2.3. Chemical Analysis

The salinity of the brine collected at end-point (day 63 from Producer 6 and day 91 from the other producers) from the middle layer of the containers, was determined by following AOAC International official volumetric method 937.09 for measuring salt (chlorine as sodium chloride) in seafood [19]. The pH was measured in brine collected from the middle layer of the containers with a pH meter (pH 1000 L, VWR, Leuven, Germany). 

### 2.4. Culture-Dependent Microbial Characterization

The growth of mesophilic, fastidious anaerobic bacteria, LAB, *Enterobacteriaceae* and yeasts were enumerated using Plate Count Agar and Blood Agar (PCA; Oxoid Ltd., ThermoFisher Scientific, Waltham, MA, USA. BA; Blood agar base and defibrinated horse blood; Oxoid and ThermoFisher Scientific), deMan, Rogosa and Sharpe agar (MRS; Oxoid), Violet Red Bile agar with Glucose (VRBG; Oxoid) and Dichloran Rose Bengal Chloramphenicol agar (DRBC; Oxoid), respectively. PCA plates were incubated aerobically for three days at 20 °C. MRS and BA plates were incubated anaerobically for five days at 20 °C and three days at 20 °C, respectively. Anaerobic incubation was performed using the AnaeroGen Atmosphere Generation System (Oxoid). VRBG and DRBC plates were incubated aerobically for two days at 30 °C and 5 days at 25 °C, respectively. 

The samples collected from the mid-level of the containers of days 0, 3, 7, 28, 91 (or day 63 from Producer 6) were subjected to microbial plating. (For the Producers 4, 5 and 6 only the end samples were collected and thus plated the first year). Samples of frozen fermented fish brine were thawed and 50 µL of non-diluted or appropriate 10-fold dilutions (using peptone water) were spread on agar plates using an automated spiral plater (Don Whitley Scientific Limited, Shipley, UK).

### 2.5. Culture-Independent Microbial Characterization

#### 2.5.1. DNA Isolation from Rakfisk Brine

Total bacterial DNA from thawed *rakfisk* brine was isolated by using DNeasy 96 Protocol (Qiagen, Hilden, Germany) in combination with mechanical lysis. Bacterial cells were pelleted by centrifugation at 15,000× *g* for 15 min, using 0.5 mL brine collected at days 14, 28, 42, 63, 91 and 4 mL brine collected at days 0, 3, 7 (to compensate for less bacterial cells early in the process). The bacterial pellets were washed twice in TES (10 mmol L^−1^ Tris-HCl pH 8.0, 1 mmol L^−1^ ethylenediaminetetraacetic acid (EDTA) pH 8.0, 100 mmol L^−1^ NaCl) with subsequent centrifugation at 15,000× *g* for one minute and resuspended in 500 μL 2 × TE-buffer (20 mmol L^−1^ Tris HCl pH 8.0, 2 mmol L^−1^ EDTA pH 8.0) with 1.2% Triton X-100. We added 8.4 ng of an external standard (plasmid containing a random DNA sequence not found in nature) [20] to normalize for eventual DNA loss related to the isolation process. The samples were then transferred to FastPrep tubes containing 250 mg glass beads (≤106 µm) (Sigma, Steinheim, Germany) and homogenized for 2 × 40 s at 4.0 m s^−1^ (FastPrep-24™ Instrument from MP Biomedicals, Santa Ana, CA, USA). Thirty five µL proteinase K and 280 µL AL-buffer (Qiagen) was added and the samples were mixed before incubated at 56 °C for 10 min. Four hundred µL of the solution was transferred to a collection microtube and the further procedures were done according to DNeasy 96 Protocol step 5 (for animal blood or cells). The DNA was eluted in 100 µL of buffer and re-eluted with the first eluate to increase the DNA concentration.

#### 2.5.2. Microbiota Analyses Using a Direct Dideoxy Sequencing Method

PCR was performed on mixed bacterial DNA using primers covering the V3 and V4 region of the 16S rRNA gene (corresponding to nucleotide positions 331 and 797 of *Escherichia coli* 16S rRNA gene) forward primer; 5′-TCC TAC GGG AGG CAG CAG T-3′ and reverse primer; 5′-GGA CTA CCA GGG TAT CTA ATC CTG TT-3′ [21]. The PCR mixture contained: 1× Dynazyme Hot start buffer (Thermo Fisher Scientific Inc., Finnzymes, Vantaa, Finland), 200 µmol L^−1^ dNTP, 0.2 µmol L^−1^ of each primer, 0.6 U Dynazyme Hot Start DNA polymerase II (Thermo Fisher Scientific Inc., Finnzymes, Vantaa, Finland), and 1.0 µL template DNA in a 25 µL reaction volume. The PCR conditions were 94 °C in an initial 10 min step, then 30 cycles of 94 °C for 30 s, 60 °C for 30 s, 72 °C for 45 s, followed by a final extension of 72 °C for 7 min and subsequently cooling to 4 °C. To remove excess nucleotides and primers, the PCR products were purified using 0.4 µL ExoSap-IT (affymetrix USB, Santa Clara, CA, USA) to 5 µL PCR mixture. Thermal conditions were 37 °C for 30 min, 80 °C for 15 min and then cooling to 4 °C. The sequencing PCR was performed using 0.32 µmol L^−1^ of the same forward primer as in the PCR reaction, 0.75× Big Dye buffer (Applied Biosystems, ThermoFisher Scientific, Waltham, MA, USA), 1 µL Big Dye 1.1 (Applied Biosystems), 1 µL purified PCR-product in a 10 µL reaction volume. The sequence reaction was performed by 25 cycles of 96 °C for 15 s, 60 °C for 4 min and subsequently cooled to 4 °C. Precipitation of the sequence products was performed using BigDye X-Terminator Purification Kit (Applied Biosystems) according to the instructions supplied by the manufacturer. Sequencing was performed on ABI 3130xl Genetic Analyzer (Applied Biosystems). A control sample, with DNA from a pure culture of *Lactobacillus sakei* 23K [22], was also amplified and sequenced using the same parameters as above. 

The mixed DNA sequence spectra were pre-processed using a script designed by [23] that works in MatLab (MathWorks, Natick, MA, USA). The pre-processed DNA sequence spectra were analyzed using principal component analysis (PCA). Multivariate curve resolution (MCR) with alternating least squares (MCR-ALS) was used to determine the pure sequence spectra (and their relative amount) of the dominant bacteria within the mixed sequences of each sample [23]. The order of nucleobases in the pure spectra was identified by a program designed by Avershina et al. [24]. Homologies to other known sequences were determined by use of the Ribosomal Database Project (RDP) Classifier [25] Naïve Bayesian rRNA classifier version 2.5 [26].

#### 2.5.3. MANOVA

Variance analyses were performed on the MCR components by using the 50–50 MANOVA (multivariate analysis of variance) software [27], where the dimensionality of the data are reduced by principal component decompositions. 

#### 2.5.4. Microbiota Analysis Using Illumina Sequencing

Selected DNA samples (end point samples from year 1 and 2 from producers 1, 2 and 3) were analyzed with Illumina sequencing using the MiSeq platform (Illumina Inc., San Diego, CA, USA). PCR was performed in triplicates and paired end sequencing (2 × 150 bp) performed using the protocol by Caporaso et al. [28]. Briefly, the V4 region of the 16S rRNA gene was amplified with region-specific primers that included the Illumina flowcell adapter sequences. The reverse amplification primer also contained a twelve base barcode sequence that supports pooling of different samples. 

Samples were purified with AMPure (Beckman Coulter, Indianapolis, IN, USA) and quantified using the Quant-iT Picogreen ds DNA with picogreen (Invitrogen, ThermoFisher Scientific, Waltham, MA, USA) before pooling. The sample pool was purified again with AMPure, quantified using the Quant-iT Picogreen ds DNA Assay (Invitrogen), diluted to 4 nM, and the MiSeq protocol “Preparing DNA Libraries for Sequencing on The MiSeq” provided by Illumina was then followed. The MiSeq Control Software (MCS) version used was RTA 1.17.28. The forward and reverse reads were joined in QIIME (version 1.7.0) and the barcodes corresponding to the reads that failed to join were removed. The sequences were then demultiplexed in QIIME allowing zero barcode errors and a quality score of 30 (Q30). 

Reads were assigned to their respective bacterial id using an open-reference OTU (operational taxonomic unit) picking protocol using the QIIME toolkit [29], where uclust [30] was applied to search sequences against the Greengenes gg_13_8_otus version. In an open-reference OTU picking process, reads are clustered against a reference sequence collection and any reads which do not hit the reference sequence collection are subsequently clustered de novo.

Beta diversity (the change in species composition across the data) was calculated by using UniFrac distance method and visualized by Principal coordinate analysis (PCoA) [31].

#### 2.5.5. Total Number of Bacteria Using Quantitative Real-Time PCR

In order to estimate total number of bacteria in a culture-independent manner, quantitative real-time PCR (qPCR) was performed on DNA isolated from *rakfisk* brine (pelleted cells, see above) of selected samples, containing a known amount of externally added reference DNA [20] using the same primers targeting the V3 and V4 regions as for direct dideoxy sequencing in addition to the probe (6-FAM)-5′-CGT ATT ACC GCG GCT GCT GGC AC-3′-(TAMRA) [21]. Standard curves were created using a known concentration of the 16S rRNA gene product and the externally added reference DNA. An internal reference dye (ROX, 6-carboxy-X-rhodamine) was used to normalize the emission from the reporter signal. We used the same concentrations of reagents and template as for the V3/V4 PCR reaction presented above, in addition to 0.1 µmol L^−1^ probe and 1× Rox (Invitrogen). For the externally added reference DNA we used forward primer: 5′-TAC CTC TAA AAT GGA TGC GCA AA-3′, reverse primer: 5′-CAC ATT CTC CTT TCG CAC GTT-3′ and probe: (6-FAM)-5′-AGC CGC CGC TTG CGA TTT AGA CC-3′-(TAMRA). The real-time PCR conditions were: 94 °C for 10 min, then 40 cycles of 95 °C for 30 s and 60 °C for 1 min. 

### 2.6. 16S rDNA Sequencing of LAB Isolated from MRS Agar

LAB isolated from MRS agar was identified by partial 16S rRNA gene sequencing (primers covering V1 and V2, 27F (5′-AGA GTT TGA TYM TGG CTC AG-3′) 907R (5′-CCG TCA ATT CMT TTG AGT TT-3′)) [32], directly on colonies using a variation of a yeast colony PCR method [33,34] as follows: Less than 0.5 µL cells (very small quantity) from fresh LAB colonies were collected from MRS agar plates and transferred to a 0.2 mL semi-skirted 96 well microtiter plate for PCR (Thermo Scientific) and the plate was sealed with strips. The PCR plate was then heated at max power for one minute in a microwave oven before adding PCR mastermix to each well. The PCR mixture contained: 1× 5 Prime Hot Mastermix (5 PRIME, Inc. Gaithersburg, MD, USA) and 0.2 µmol L^−1^ of each primer in a 12.5 µL reaction volume. The PCR conditions were 9 °C for 2 min in an initial activation step, then 35 cycles of 94 °C for 30 s, 50 °C for 30 s, and 72 °C for 1 min, followed by a final extension of 72 °C for 7 min, and subsequently cooled to 4 °C. The PCR products were then analysed by agarose gel electrophoresis and the PCR products were diluted 1:10 before sequencing PCR. The ExoSap-IT reaction to clean up the PCR product, the sequencing PCR reaction, the precipitation of PCR products and the sequencing was done as described in Section 2.5.2 with 27F as sequencing primer. Sequence homologies were determined by use of the SequenceMatch [26,35].

## 3. Results

### 3.1. Chemical Measurements

**Figure 1:**
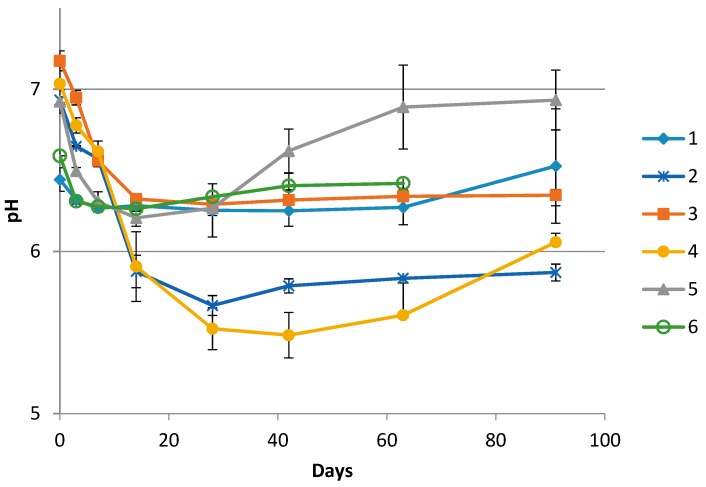
pH values in brine collected from the middle layer in the containers from the individual producers (1–6) during the *rakfisk* ripening process. (Day 63 was the last sampling day from Producer 3). Error bars represents the standard deviation of the triplicate biological replicates.

The pH development for one complete sampling season for all producers is shown in Figure 1. The initial pH values of the *rakfisk* brine samples were between 7.2 and 6.4. During the first days the pH values decreased to varying extent in all the productions. However, the largest reductions were seen in productions where sugar was added (Producer 2 and 4). After the initial decrease, the pH values were relatively stable throughout the ripening period or increased towards the end. The increase was most evident for Producer 5. For the producers where a second season sample series was available (Producers 1, 2, and 3), the pH profile appeared essentially the same. The salinity ranged between 3.5% and 7.4% NaCl (*w/v*) from the different producers and years (Table 1). 

### 3.2. Plate Counts and Total Number of Bacteria Using qPCR

**Figure 2:**
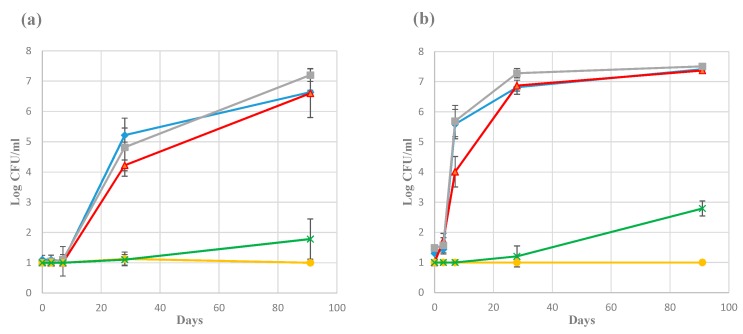
Microbial growth in brine during *rakfisk* ripening from the first year from Producer 1 (**a**) and Producer 3 (**b**). Error bars represents the standard deviation of the triplicate biological replicates. The detection limit was 20 CFU/mL. 10 CFU/mL were used as default number from the plates where no growth could be detected. ■ (grey), BA; ♦ (blue), PCA; ▲ (red), MRS; x (green), VRBG; ● (yellow), DRBC (Dichloran Rose Bengal Chloramphenicol).

Figure 2 shows the development of the plate count bacterial numbers in the brine (mid-level samples) collected from Producers 1 and 3 for one season. This represents the productions with the slowest and fastest bacterial growth, respectively, and serve as examples from the complete dataset for all producers (not shown). Small differences were also observed between numbers obtained from either PCA, Blood agar, or MRS agar. Similar development as for Producer 3 was seen for the Producers 2, 4, and 5, especially from day 28 and onwards. Producer 6, with a slightly different manufacturing procedure (see Section 2.2 and Table 1), had total counts between 10^6^ and 10^7^ CFU/mL throughout the sampling period. For samples from the corresponding time-points the second season (complete series for Producers 1, 2, and 3; end-point samples for Producer 4, 5, and 6), the bacterial numbers appeared essentially the same as for the first season. The addition of starter culture by Producer 4 was evident by high numbers (approximately 10^5^–10^6^ CFU/mL) in the day 0 samples. There were no significant differences between samples from the different layers of the same container. Some growth on VRBG (enterobacteria) and/or DRBC (yeast) was evident for most producers, but with low numbers (up to 10^3^ CFU/mL). However, growth on these media was not detected in any of the samples from Producer 2 and 4.

The bacterial 16S rRNA gene copy numbers per mL brine, measured by qPCR, were determined in samples from day 3, 14, and end point (day 63 from Producer 6, day 91 from all other producers). At day 3, the numbers were 10^3^–10^4^ copies per mL brine for producers 1 and 3, and 10^5^–10^6^ copies per mL brine for all other producers. At day 14, the copy numbers had increased to 10^7^–10^8^ per mL brine from all producers. The end point samples from all producers had copy numbers of approximately 10^8^ per mL brine.

### 3.3. Microbiota Analysis Using Culture-Independent Characterization

#### 3.3.1. Direct Dideoxy Sequencing

One thousand seven hundred and ten (1710) samples from the entire sampling period from two years were sequenced by a direct dideoxy sequencing approach using primers covering the V3 and V4 area of the 16S rRNA gene. Mixed sequence spectra were obtained from 1342 of them. (The remaining 368 samples, mainly from early time-points (day 0 and day 3), were not successfully sequenced due to too little or poor quality of DNA). 

An initial PCA analysis of the pre-processed DNA sequence spectra suggested eight components to be resolved by MCR-ALS (which would explain 92% of the variance in the data set). However, two of the components contained the same information, so biological reasoning would suggest using fewer components. In addition, when applying fewer components, the control samples (*Lactobacillus sakei* 23K) were predicted with slightly higher correlation (Pearson’s correlation between actual and predicted values) and the explained variance was still very high. Finally, five components were chosen, accounting for 90% of the variation in the data. These components were resolved by RDP classifier. The lowest taxonomic rank with a confidence threshold above 80% was used. The resolved components were identified to different taxa: Components 1; class *Gammaproteobacteria*, 2; order *Bacillales*, 3; order *Lactobacillales*, 4; genus *Psychrobacter*, 5; genus *Lactobacillus* (Supplementary Table S1).

The five MCR-ALS components were analyzed by using 50–50 MANOVA and the largest variation was due to differences between the producers. However, there were also small, but significant, differences between the years. The producer and year interaction means that the year variation differs between the producers (Table 2). The R^2^-values (meaning the measure of how much of the variation that is explained by the model) were: *Gammaproteobacteria* = 0.64; *Bacillales* = 0.56; *Lactobacillales* = 0.37; *Psychrobacter* = 0.84; *Lactobacillus* = 0.81.

**Figure 3:**
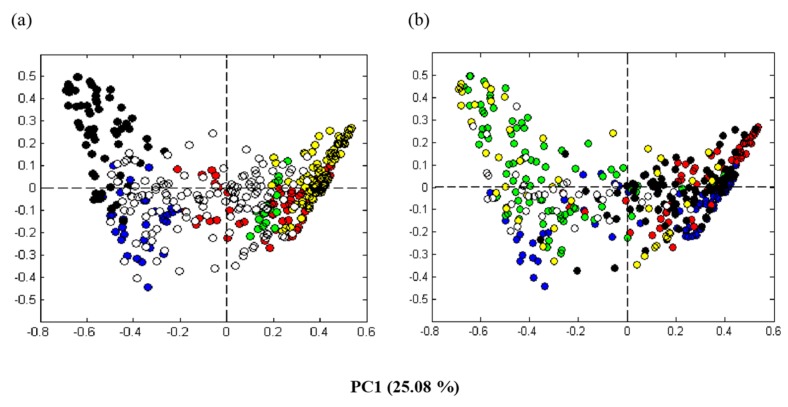
Score plots of the principal component analysis (PCA) for the pre-processed aligned partial 16S rRNA gene spectra. *Rakfisk* brine samples were collected from the six producers throughout the ripening period during two years (see Table 1). (**a**) The data points visualized according to the bacterial taxa corresponding to the dominating MCR (multivariate curve resolution) component of each sample; blue: *Gammaproteobacteria* (component 1), red: *Bacillales* (component 2), green: *Lactobacillales* (component 3), black: *Psychrobacter* (component 4), yellow: *Lactobacillus* (component 5), and white: none of the components were above 50%. (**b**) The data points visualized according to the six different *rakfisk* producers; 1: green, 2: blue, 3: black, 4: red, 5: yellow, 6: white.

The average values of the pre-processed DNA sequence spectra from the triplicate sampling of each level (top-, mid-, and bottom-level of each container) were analyzed by PCA. The two first components, accounting for 33% of the total variance in the data, were plotted. To visualize factors that might be separating the data, the sample points in the PCA plot were colored according to different variables (i.e., dominating MCR components, producers, sampling days, ripening temperatures, and salt concentrations). (When one sequence type accounted for more 50% of the of the sequence types in a given sample, the corresponding MCR component was designated as the dominating MCR component). Visualizing the samples according to the dominating MCR components showed that samples located to the left side of the PCA plot were dominated by bacteria belonging to the class *Gammaproteobacteria* (components 1 and 4) and samples located to the right side were dominated by bacteria belonging to the class *Bacilli* (components 2, 3, and 5) (Figure 3a). Visualizing according to the different producers appeared complex. Nonetheless, there seemed to be some trends in which the majority of samples originating from the individual producers were dominated by *Gammaproteobacteria* (Producers 1 and 6) or *Bacilli* (Producers 3 and 4). Regarding the Producers 2 and 5, some of the samples were dominated by *Gammaproteobacteria* while other were dominated by *Bacilli* (Figure 3b). For Producer 2, this was due to a clear difference between early and late samples (see also below). Producer 5 had the largest sample variation in general, with no clear tendencies (Figure 3b). 

**Figure 4:**
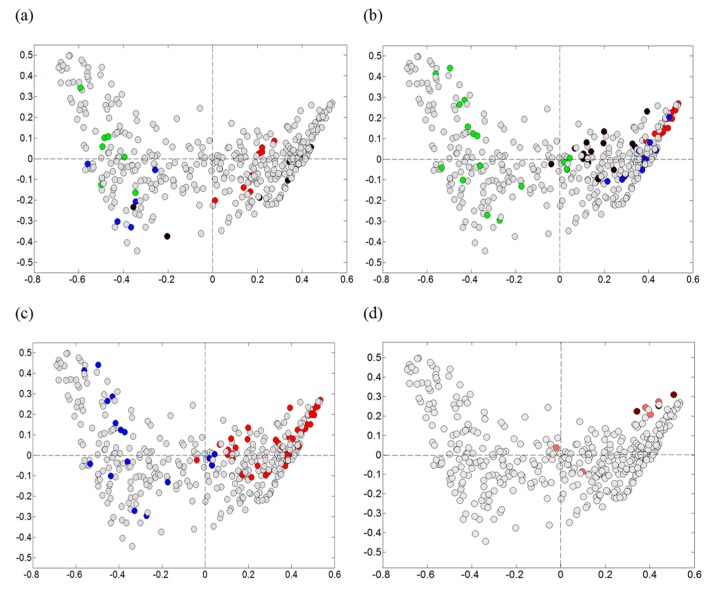
Visualization of early/late samples and salinity/ripening temperature. Showing the same PCA plot as in Figure 3, however visualizing by colors the mid-level samples from four of the producers (all other data-points are grey). Samples collected (**a**) early (days 0, 3, and 7) and (**b**) late (days 42, 63, and 91) during the *rakfisk* ripening from the producers; 1: green, 2: blue, 3: black, 4: red. (**c**) Blue: high salinity (≥5.8% NaCl) combined with low ripening temperature (≤4.5 °C) (Producer1), red: low salinity (≤5% NaCl) combined with higher temperature (≥5 °C) (Producers 2, 3, and 4). (**d**) Including data points from an additional experiment where Producer 1 prepared their regular fish using a lower salinity combined with a higher ripening temperature. Pale red; day 42 and 63, dark red: day 91.

Visualization according to sampling times were complex and difficult to interpret. To obtain a more clear depiction of the development of the microbiota, we chose to visualize only the mid-layer sample points from the producers applying a more standardized process line production (Producers 1, 2, 3, and 4), in which the raw materials from batch to batch had a greater probability of being subjected to equal handling (Figure 4). We could see a dynamic between samples collected early (Figure 4a) and late (Figure 4b) during the ripening, with a clear shift in the microbiota in samples from Producer 2. The samples collected late (days 42, 63, and 91) from the Producers 2, 3, and 4, were dominated by *Bacilli*. The corresponding samples from the Producer 1 were mainly dominated by *Gammaproteobacteria*. Producers 2, 3, and 4 used lower salinities (≤5% NaCl) combined with higher ripening temperatures (temperature ≥5 °C), compared to Producer 1 (Figure 4c). In the additional experiment, where Producer 1 made a change in the salinity (from 5.8% NaCl to 4.5% NaCl) and in the ripening temperature (from 3.5 °C or 4.5 °C to 7 °C), the microbiota were then directed towards *Bacilli* (Figure 4d) with the late samples dominated by *Lactobacillus* (see also below).

Since *Psychrobacter* and *Lactobacillus* were the two genera that best separated the producers in the PCA analysis (Figure 3a) and had the largest R^2^-values from the 50–50 MANOVA analysis (see above), we chose to specify the relative amounts of these two genera in the early samples and in the late samples from all the producers (as well as from the additional experiment where Producer 1 changed the normally applied salinity and temperature). *Pychrobacter* dominated in the late samples from the producers 1 and 6, while *Lactobacillus* dominated from the producers 2, 3 and 4. In addition, late samples from the additional experiment by Producer 1 were dominated by *Lactobacillus*. A significant increase of the relative amount of *Lactobacillus* was seen in all the productions where a low salinity (≤5% NaCl) was combined with a higher temperature (≥5 °C), except for Producer 5. *Psychrobacter* and *Lactobacillus* each accounted for about 25% of the total bacterial load in the late samples from Producer 5 (Table 3). 

#### 3.3.2. Illumina (MiSeq) Sequencing

**Figure 5:**
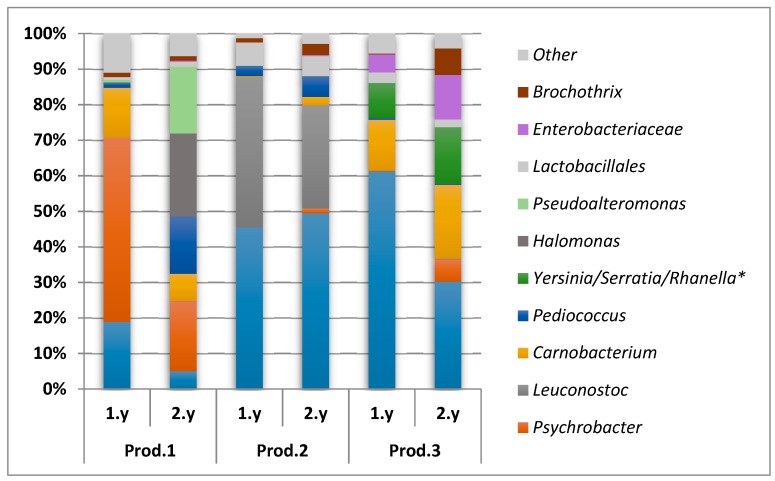
Dominating microbiota (all taxa above 1% across all samples) obtained from Illumina (MiSeq) of brine samples collected day 91 from the first and the second year (1.y and 2.y, respectively) from the Producers (Prod.) 1, 2, and 3. * Not possible to distinguish between these genera in the analysis.

In order to validate the direct dideoxy sequencing results and to achieve a higher resolution of the microbiota, high-throughput sequencing (Illumina, MiSeq) of the V4 region of the 16S rRNA gene were conducted for a selection of samples. In total, 12 samples collected on day 91 during two subsequent years from the two largest producers not applying starter culture, Producers 1 and 3, were selected. In addition, six equivalent samples from Producer 2 were included to confirm the presence of *Leuconostoc*. For samples from this producer, *Leuconostoc* was seen in the direct dideoxy sequencing when applying eight components in the MCR-ALS analysis, but was not included as a separate group when applying fewer components (see above). In total, 65 bacterial genera and one archaeal genus were identified. However, only eight genera, two unclassified families and one unclassified order constituted above 1% of the total bacterial load of the average values for the biological triplicates across all samples (Figure 5). These bacterial taxa belong to the classes *Gammaproteobacteria* and *Bacilli*. The microbiota in the brine from Producer 2 was dominated by LAB (in this context: *Lactobacillales, Lactobacillaceae, Leuconostoc, Carnobacterium,* and *Pediococcus*) and consisted of substantially equal proportions of *Lactobacillaceae* and *Leuconostoc*. The microbiota in the brine from Producer 3 were also dominated by LAB and even though the proportion of LAB relative to *Gammaproteobacteria* was lower the second year, the largest dominating microbial groups were present both years. The microbiota in the brine from Producer 1 was dominated by *Gammaproteobacteria* both years; *Psychrobacter* accounted for a large amount the first year, while a more equal distribution between *Psychrobacter*, *Halomonas,* and *Pseudoalteromonas* were seen the second year. The proportion of LAB was approximately the same both years (Figure 5).

**Figure 6:**
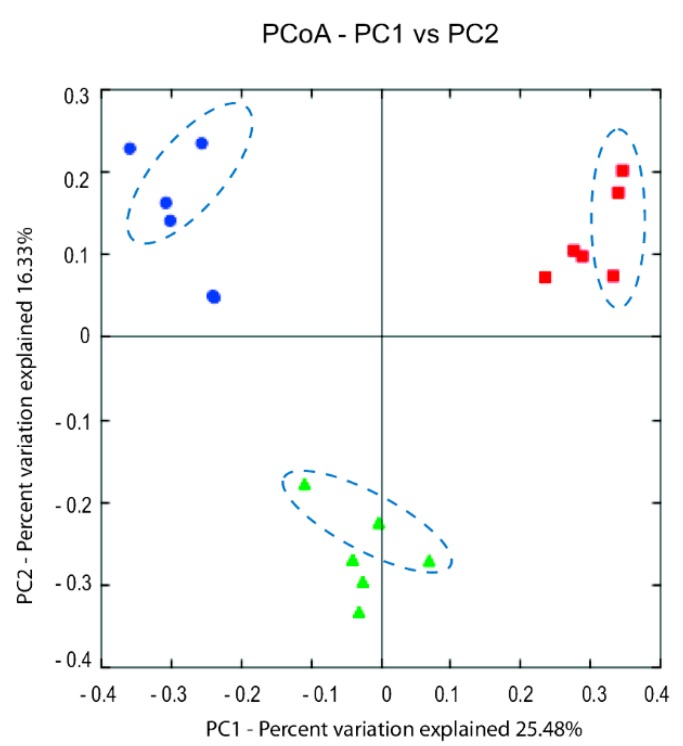
PCoA (Principal coordinate analysis) plot visualizing the unweighted UniFrac diversity between samples collected day 91 from the three producers: 1; red squared shapes, 2; blue round shapes, 3; green delta shapes. The encircled data points represents samples collected from the second year. PC1 and PC2, principal component 1 and 2, respectively.

Beta diversity analysis showed the samples analyzed with MiSeq sequencing were separated based on the producers using the both unweighted- and weighted-UniFrac distance matrix. The PCoA plot (unweighted) (Figure 6) show three distinct clusters corresponding to the three producers and that the year to year variation within the individual producers were small. However, the weighted-UniFrac distance matrix showed that the year to year variation were higher for Producer 1 compared to the other two (Supplementary Figure S1). This indicate that the dominating microbiota shifted between the two corresponding years for this producer (in accordance with the results shown in Figure 5) and correlate to the fact that this producer changed the ripening temperature from 3.5 °C the first year to 4.5 °C the second year.

### 3.4. Species Determination of LAB Isolated from MRS Agar

In order to obtain an indication of which species were dominating the LAB community, we performed a partial 16S rDNA sequencing on pure colonies isolated on MRS agar from end-point samples of all producers (except from Producer 4, who used a *Lactobacillus sakei*-based starter culture). From a total of 188 colonies, 155 were successfully sequenced (sequences shorter than 518 bases were discarded) and classified by use of SequenceMatch (RDP). Of these, 120 (77%) showed 100% sequence similarity to the corresponding sequences of the type strains of *L. sakei* subsp. *sakei* and *L. sakei* subsp. *carnosus*. Other species identified were *L. curvatus* (6), *Carnobacterium maltaromaticum* (5), *C. divergens* (1) and *Streptococcus salivarius* (1). The remaining sequences (22) were not unequivocally classified, but belonged to the *Lactobacillus* or *Leuconostoc* genera.

## 4. Discussion

This work represents the first study of microbiota in the traditional Norwegian fermented fish product, *rakfisk*. The nature of the production and manufacture of *rakfisk* poses some logistic challenges for microbiological analysis and it was inevitable that some compromises with an ideal situation had to be made. First, sampling of real production batches of *rakfisk* had to be done by collecting brine instead of the actual fish. The fish is tightly packed or pressed, meaning that any withdrawal of fish from a representative part of a container (i.e., the center) will disturb and potentially spoil the process (e.g., by changing oxygen access) in that particular container. A full sampling series of fish would thus require that a large number of containers were produced in parallel, with the “sacrifice” of containers at each time-point. This was considered logistically and economically impossible for such small enterprises that *rakfisk* producers represent. We therefore designed a brine sampling procedure, which allowed sampling from the same container at different time points without disturbing the process. Earlier, preliminary studies [13] also indicated that microbiological analyses of brine samples were reflecting actual fish samples. Second, the producers are located at remote areas of the country, far from the research facilities. Brine samples were therefore taken by the producers themselves, frozen, and accumulated during the entire sampling period. The total sample set (frozen) was subsequently sent to the laboratory. Freezing of samples may not be optimal with regard to either classical microbiological analysis, or before DNA preparation from pelleted samples, due to the possible death and lysis of bacteria during freezing/thawing, thereby introducing a bias. Control experiments were performed to evaluate the importance of the effects of this freezing procedure (see further below).

The microbiota obtained from the different producers towards the end of the ripening were dominated by bacteria belonging to the classes *Gammaproteobacteria* and *Bacilli*, and the largest variance was associated with the genera *Psychrobacter* and *Lactobacillus*, respectively. Compared to the DNA-based microbiota analysis, limited information was obtained from the classical microbiological analysis. The variation in the material due to *Gammaproteobacteria*/*Psychrobacter* and *Bacilli*/*Lactobacillus* was not evident in this analysis using standard media. However, the initial results obtained by direct sequencing were corroborated by Illumina sequencing (MiSeq) of selected samples. Most of the variance could be explained by sample origin, i.e., the different producers. The microbiota was to a large extent reproducible in the two consecutive productions of each producer. It appeared that relatively small differences in the salinities and the ripening temperatures determined the microbiota profiles. This was supported by the results from the small additional experiment performed by Producer 1, where the change to a combination of a lower salinity and a higher ripening temperature directed the microbiota to the domination of *Lactobacillus* instead of *Psychrobacter* (and/or other *Gammaproteobacteria*) in the late samples (Figure 4d). Even the slight change of temperature from year 1 to year 2 (3.5 °C to 4.5 °C) by Producer 1, clearly induced a change in the microbiota (Figure 5). Of the producers, Producer 5 appeared to have the most variable process (Figure 3b), possibly a result of relatively low selective pressure (low salt, high temperature), less standardized raw material (wild-caught fish) and the largest difference in salt concentration from the first year to the next (Table 1). 

The dominance of *Gammaproteobacteria* and/or *Bacilli* was established early in the ripening process. The *Bacilli* were mainly comprised of LAB. Most fermented food is dependent on LAB to mediate the fermentation process and LAB contributes to alteration of flavor, odor, and texture as well as preservation [2]. LAB have also been found to dominate or be present in high numbers in several other types of fish fermentations [6,7,8,9,10,11]. LAB were found in samples from all the producers and the majority of the colonies isolated from MRS agar were identified as *L. sakei*. This species is often associated with various meat fermentations [36] and one of relatively few LAB able to grow in the salt concentrations and the ripening temperatures applied during *rakfisk* fermentations [37,38]. *L. sakei* was also found to be the dominant LAB in *sikhae* and *gajami-sikhae,* mild-salted fermented fish products from Korea [15]. The Chinese product *Suan Yu*, with some similarities to rakfisk fermentation [10], but fermented at higher temperature, was shown by microbiota analysis to be quickly dominated by *Firmicutes* and lactobacilli with higher temperature preferences, such as *L. plantarum* [39]. *Rakfisk* from Producer 2 appeared to have a co-dominance between lactobacilli and leuconostocs. This producer applied the lowest salt concentration of the producers in the manufacture, which could affect the selection of fermentative bacteria. However, also the raw material from this producer differs from the others (land-based farmed char). Co-fermentation by leuconostocs and lactobacilli are common in other food fermentations, especially vegetable fermentations such as sauerkraut or kimchi [40].

Compared to LAB, there are few studies reporting *Gammaproteobacteria* to be dominating in fermented food. This taxon has been found dominant in shellfish [18] and in cured skate [41]. However, several studies found *Gammaproteobacteria* as a non-dominant part of the bacteria in fermented food [42,43,44] or found that the domination of this taxon has been replaced by *Firmicutes* towards the end of the fermentation [15,45,46]. In our study, *Gammaproteobacteria* was dominating in the brine when *rakfisk* was produced with a high salinity combined with a low ripening temperature. The majority of the bacteria in this class belonged to the genus *Psychrobacter. Psychrobacter* species are ubiquitous in the environment and can be isolated from a number of habitats [47]. The psychrotrophic nature make them competitive at low temperatures and they can be isolated from processed, cold-stored meat and poultry products in addition to fish and fish products, in many cases as spoilage organisms [48]. *Psychrobacter* has been reported to be aerobic [49,50,51,52]. However, Bjørkevoll et al. [53] found *Psychrobacter* species in salted cod which were facultatively aerobic. *Psychrobacter* species are halotolerant, have a lower growth optimum temperature than for instance *L. sakei* [54,55] and are therefore suited to grow in *rakfisk* brine. Interestingly, *Psychrobacter* was found to be the third most dominant genera (at 15%) after fermentation in the aforementioned Korean fermented fish product *sikhae,* dominated by *L. sakei* [15]. Notably, a *Psychrobacter* strain has been used as a starter culture for fish sauce fermentation due its proteolytic properties [56]. However, the fermentation process of fish sauce is very different from *rakfisk* (e.g., saturated salt concentration and high temperature) and the actual role of *Psychrobacter* in *rakfisk*, or similar, fermentations for the character of the final product remains unknown.

The lowest pH values and the highest proportions of LAB were detected in the brine from *rakfisk* productions where sugar was added (Producer 2 and 4). These conditions may initially select for LAB, which lower the pH by lactic acid production, further amplifying selection towards LAB. This could be advantageous from a food safety perspective. The fact that enterobacteria and yeasts were not detected for these producers in the classical microbiological analysis strengthen this notion. Although pH rose later during the process, end-pH was still lower for these producers compared to the others. Addition of starter culture (as performed by Producer 4) could also be an advantage for the same reason, although it is not known at present if the *L. sakei* strain present in the starter actually dominated the fermentation. 

Although the microbiota in the brine differed significantly between the producers, and their products normally have some sensory differences, the different ripening processes nonetheless resulted in the distinctive product: *rakfisk*. This suggests that factors other than the microbiota composition and the resulting activity might be important in defining this product. The concept of “autolysis”, i.e., degradation of fish proteins and lipids by endogenous enzymes, was already suggested to be important for the character of the product in very early investigations of *rakfisk* [14]. There are some indications that the fermentation phase (growth of the bacteria) seems to be finished several weeks before the product is mature, i.e., the development of the number of bacteria, as determined by plate count (Figure 2b) and by 16S rRNA gene copy numbers (Section 3.2). This may indicate that autolysis is indeed important in the later stages of maturation. Further investigations are needed to explain the roles of autolysis and bacterial fermentation for the taste and texture development of *rakfisk*.

As mentioned, the samples had to be frozen by each producer before analysis due to logistic considerations. The disadvantage of this procedure could be that some bacteria may not grow well after freezing [57] and/or that some cells may have lysed during the freeze/thawing process. However, the differences in the bacterial numbers obtained by culturing methods and qPCR for the end-point samples (both analyzed on the cell pellet after centrifugation), suggest that the bacterial cells did not lyse to a large degree during freezing. Dead cells could also be a relevant problem in microbiota analysis based on DNA [58]. The fact that qPCR may overestimate the bacterial load with as much as one order of magnitude due to multiple rRNA gene copies per chromosome and more than one chromosome per cell [59,60], suggests that dead cells did not contribute substantially to the total DNA amount in our study. Differences between plate count and qPCR were, however, larger in early samples with qPCR giving much higher estimates. The reasons for this are unknown at present.

As a control, we also performed bacterial community analysis using MiSeq on selected supernatants (end-point samples) after the normal centrifugation for pelleting cells. DNA was present in these supernatants, indicating a certain amount of lysis of cells, which could originate from freezing/thawing or from natural lysis during the maturation period. However, the microbiota profiles of these samples was essentially the same as from the pellet of the same sample (results not shown), indicating that minimal bias was introduced during the freezing/thawing, at least not for the dominating taxa. This would suggest that the results obtained to a large degree reflect the true microbiota development in *rakfisk* production. Freezing of samples before microbiological analysis has in some cases also been shown to be of minor importance for complex (and presumably sensitive) fecal samples [61]. Rather, rapid freezing can be a procedure to minimize the introduction of other factors contributing to bias, such as time for transport and different storage times at higher temperatures.

## 5. Conclusions

In conclusion, the microbiota in the brine from the six *rakfisk* producers was dominated by *Gammaproteobacteria* and LAB, mainly represented by the genera *Psychrobacter* and *Lactobacillus*, respectively. The individual producers had a relatively reproducible microbiota development from one year to the next. Variations in the microbiota have implications for understanding how the *rakfisk* ecosystems are affected by environmental factors. The microbiota appears to be determined by the relatively small differences in the salinity and the ripening temperature. However, further studies applying more controlled experiments would be needed to determine the significance of different salinities combined with different temperatures.

## Figures and Tables

**Table 1:** Different parameters used during the production of *rakfisk* in this study. (The temperatures were measured and given by the individual manufacturers).

Producer ID	Fish Species/Origin ^b^	Temperature °C	NaCl % (*w/v*) ^d^	Sugar Addition	Starter Culture Addition ^g^	Salting Method ^i^	Sampling ^k^
1 ^a^	Char/A	3.5/4.5 ^c^	5.8/5.7	No	No	Dry	Complete
2	Char/B	5.0	3.5/4.0	Yes ^e^	No ^h^	Brine	Complete
3	Trout/C	7.0	4.9/4.2	No	No	Dry	Complete
4	Trout/C	7.0	4.8/5.1	Yes ^f^	Yes	Dry	Year 2+
5	Trout/D	7.5	4.9/3.9	No	No	Dry	Year 2+
6	Trout/E	6.0	6.9/7.4	No	No	Dry	Year 2+ ^m^

^a^ Producer 1 applied higher ripening temperature (7 °C) and lower salinity (4.3% NaCl) in an additional experiment the second year. ^b^ A, ocean-based (brackish water), farmed arctic char; B, Land-based, farmed arctic char; C, land-based, farmed trout; D, wild-caught trout (lake in Western Norway); E, wild-caught trout (mountain lake in Southern Norway). ^c^ The ripening temperature given by the producer was 3.5 °C the first year and 4.5 °C the second year. ^d^ Mean values of the NaCl concentration (% *w/v*) calculated from end-point samples (middle layer). The values represent the 1st and 2nd year, respectively. Standard deviation were in all cases less than 0.01. ^e^ 0.33% sucrose; ^f^ 0.4% fructose; ^g^ T-RM-53 containing *Lactobacillus sakei* and *Staphylococcus carnosus* (Chr. Hansen, Hørsholm, Denmark); ^h^ This producer was claiming to use the T-RM-53 starter culture. However, the microbiological analyses (both culture-dependent and culture-independent) suggested this was not the case, or the level of starter addition was below detection due to errors by the producer. ^i^ Refers to how the salt was added to the fish during preparation. After the first 24 h, productions with dry salted fish were also completely covered in brine, either from natural formation, or topped up with brine holding the appropriate salinity. ^k^ “Complete” refers to full sampling series (days: 0, 3, 7, 14, 28, 42, 63, and 91) for two consecutive years; “Year 2+” refers to full sampling the second year and end-point (day 91 or 63) sample collected the 1st year. ^m^ Sample collection ended on day 63 for Producer 6.

**Table 2:** Explained variance and significance by 50–50 MANOVA (multivariate analysis of variance) for the MCR (multivariate curve resolution) components based on samples collected days 63 and 91 from the mid-level of the containers.

Category	Explained Variance (%)	*p*-Value
**Producer ^a^**	45.43	<0.0001
**Year ^b^**	6.98	0.0001
**Producer and year ^c^**	16.99	<0.0001
**Error**	27.42	

^a^ All components were significant (*p*-value ≤ 0.05); ^b^ *Bacillales* and *Lactobacillales* were significant (p-value = 0.01); ^c^ *Gammaproteobacteria*, *Bacillales*, *Psychrobacter* were significant (*p*-value = 0.01) while *Lactobacillus* not were significant at a 5% level (*p*-value = 0.1).

**Table 3:** The relative amount (% of selected bacteria compared to the total bacterial load) of *Psychrobacter* and *Lactobacillus* in samples collected early (days 0–7) and late (days 63–91).

Producers	*Psychrobacter*			*Lactobacillus*		
	0–7 d	63–91 d	*p*-Value	0–7 d	63–91 d	*p*-Value
1	30.7 ± 20.6	50.5 ± 31.5	0.2	5.4 ± 2.9	5.7 ± 8.6	0.9
2	27.1 ± 18.3	0.5 ± 0.9	0.02	0.1 ± 0.2	80.5 ± 9.10	<0.00001 ^a^
3	2.7 ± 5.3	7. 6 ± 6.8	0.1	16.5 ± 11.1	38.3 ± 24.3	0.04 ^a^
4	5.9 ± 3.2	1.2 ± 1.0	0.02	12.4 ± 7.5	79.6 ± 11.2	<0.000001 ^a^
5	9.2 ± 18.5	25.8 ± 20.7	0.2	18.7 ± 11.1	23.4 ± 34.7	0.8
6	25.5 ± 16.8	41.6 ± 14.2	0.09	10.6 ± 13.6	7.7 ± 9.1	0.7
1 ^b^	ND	10.0 ± 4.1	0.05	ND	72.1 ± 17.2	<0.00001 ^a,c^

ND, not determined; ^a^ Producers with significant increase of *Lactobacillus* from the early samples (0–7 d) to the late samples (63–91 d). ^b^ Additional batch by Producer 1 with lower salt concentration and higher temperature; ^c^ *p*-value calculated for the difference between the late samples of the traditional production from Producer 1 and this Producer’s additional production where a lower salinity and a higher ripening temperature were applied.

## Supplementary Materials

The following are available online at http://www.mdpi.com/2304-8158/8/2/72/s1, Figure S1: PCoA plot visualizing the weighted UniFrac diversity between samples (MiSeq) collected at day 91 from the three producers: 1; red squared shapes, 2; blue round shapes, 3; green delta shapes. The encircled data points represents samples collected from the second year from Producer 1, Table S1: Classification of the MCR-ALS resolved components using the ribosomal database project (RDP) hierarchical classification. The taxonomic rank with a bootstrap confidence level (given in the brackets) above 80% (in bold typeface) was chosen to represent the corresponding components.
