# Supplementary material for: Exploring the Brine Microbiota of a Traditional Norwegian Fermented Fish Product (Rakfisk) from Six Different Producers during Two Consecutive Seasonal Productions
Source: Foods. 2019 Feb 14;8(2):72. doi: 10.3390/foods8020072 (PMC6406850; doi:10.3390/foods8020072)
Supplement: Supplementary file 1 [file foods-08-00072-s001.pdf]

Supplementary material to:

"Exploring the brine microbiota of a traditional Norwegian fermented fish product (*Rakfisk*) from six different producers during two consecutive seasonal productions"

by Bjerke et al.

Supplementary Table S1: Classification of the MCR-ALS resolved components using the ribosomal database project (RDP) hierarchical classification. The taxonomic rank with a bootstrap confidence level (given in the brackets) above 80% (in bold typeface) was chosen to represent the corresponding components.

MCR 5 components	
comp1	*Proteobacteria* [83%]; ***Gammaproteobacteria*** [80%]; *Alteromonadales* [38%]; *Alteromonadaceae* [23 %]; *Aliagarivorans* [20 %]
comp2	*Firmicutes* [100 %]; *Bacilli* [100 %]; ***Bacillales*** [87 %]; *Listeriaceae* [73 %]; *Brochothrix* [65 %]
comp3	*Firmicutes* [99 %]; *Bacilli* [99 %]; ***Lactobacillales*** [98 %]; *Lactobacillaceae* [72 %]; *Lactobacillus* [56 %]
comp4	*Proteobacteria*[99 %]; *Gammaproteobacteria* [99 %], *Pseudomonadales* [87 %]; *Moraxellaceae* [87 %]; ***Psychrobacter*** [81 %]
comp5	*Firmicutes* [100 %]; *Bacilli* [100 %]; *Lactobacillales* [100 %]; *Lactobacillaceae* [100 %]; ***Lactobacillus*** [81 %]

Supplementary Figure S1

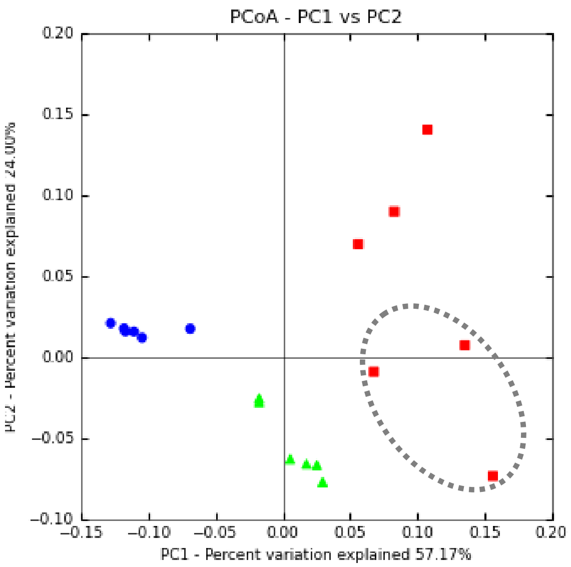

Figure S1: PCoA plot visualizing the *weighted* UniFrac diversity between samples (MiSeq) collected day 91 from the three producers: 1; red squared shapes, 2; blue round shapes, 3; green delta shapes. The encircled data points represents samples collected from the second year from Producer 1.
